# Prevalence and risk factors for acute kidney injury at the diagnosis of juvenile idiopathic arthritis in children and its long-term implications for kidney health

**DOI:** 10.1007/s00467-026-07222-9

**Published:** 2026-03-03

**Authors:** Maria Francesca Gicchino, Mattia Arenella, Caterina De Simone, Federica Di Domenico, Maria Grazia Fusco, Silvio Naviglio, Paola Tirelli, Giulio Rivetti, Anna Di Sessa, Stefano Guarino, Emanuele Miraglia del Giudice, Alma Nunzia Olivieri, Francesca Orlando, Pierluigi Marzuillo

**Affiliations:** 1 Department of Woman, Child and of General and Specialized Surgery, Università degli Studi della Campania “Luigi Vanvitelli”, Via Luigi De Crecchio 2, 80138 Naples, Italy; 2 Department of Translational Medical Science, Section of Pediatrics, University of Naples “Federico II”, 80126 Naples, Italy; 3 Department of Precision Medicine, Università degli Studi della Campania “Luigi Vanvitelli”, 80138 Naples, Italy; 4 General Pediatrics and Immuno-Rheumatology Unit, Santobono-Pausilipon Hospital, 80129 Naples, Italy; 5 Department of Pediatric Subspecialties, Nephrology and Dialysis Unit, Children’s Hospital Bambino Gesù, IRCCS, Rome, Italy

**Keywords:** Acute kidney injury, Juvenile idiopathic arthritis, Chronic kidney disease, Hypertension, Non-steroidal anti-inflammatory drugs

## Abstract

**Background:**

We aimed to investigate the prevalence and risk factors of acute kidney injury (AKI) at juvenile idiopathic arthritis (JIA) onset and its impact on long-term kidney outcomes.

**Methods:**

In this multicenter study, we retrospectively reviewed 192 children diagnosed with JIA (1998–2025). AKI was defined according to KDIGO serum creatinine criteria. Kidney damage (KD) was defined as chronic kidney disease (CKD) and/or hypertension at follow-up. Logistic and Cox regression analyses were used to identify risk factors and to calculate odds ratio (OR) and hazard ratio (HR), respectively. Kaplan–Meier analysis evaluated KD-free survival.

**Results:**

At JIA onset, 45 patients (23.4%) developed AKI, mostly stage 1, with no cases requiring dialysis. Independent predictors of AKI were younger age, elevated C-reactive protein, and ANA positivity. After a mean follow-up of 6.3 years (range 1–27.5), 23 patients (12%) developed KD (19 CKD, 4 hypertension, 2 both). Patients with AKI at onset had a significantly higher risk of KD (OR 5.8, 95% CI: 2.4–15.2; HR 3.7, 95% CI: 1.9–8.9). At 25.1 years of age, the cumulative proportion free from KD was 26.9% in patients with AKI versus 64.1% in those without (*p* = 0.005).

**Conclusions:**

AKI is relatively frequent at JIA onset and represents a strong predictor of long-term kidney damage. Early recognition and careful follow-up of children with AKI may help identify those at greatest risk for adverse kidney outcomes.

**Graphical abstract:**

**Figure:**
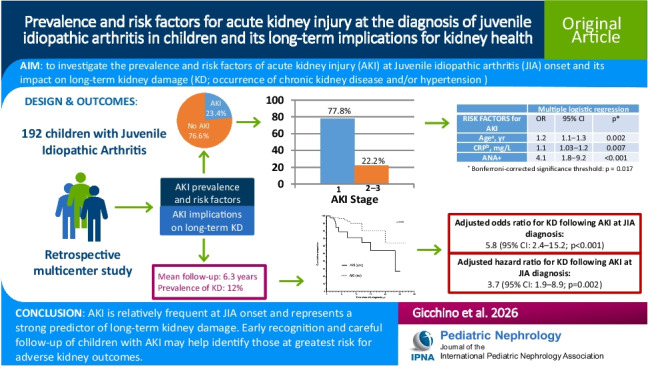
A higher resolution version of the Graphical abstract is available as Supplementary information

**Supplementary Information:**

The online version contains supplementary material available at 10.1007/s00467-026-07222-9.

## Introduction

Juvenile idiopathic arthritis (JIA) is the most common chronic rheumatic disease in childhood [1]. JIA comprises a heterogeneous group of chronic arthritis with onset before the age of 16 years, persisting for a minimum of 6 weeks, and lacking an identifiable cause [2]. Beyond articular involvement, JIA may affect extra-articular sites, including the eyes, skin, and internal organs, potentially resulting in long-term disability and, in severe cases, mortality [2].

Anecdotal reports have described kidney involvement in JIA [3–8]. The proposed mechanisms include chronic inflammation leading to renal amyloidosis; the development of secondary glomerulonephritis (e.g., membranous nephropathy, mesangial IgA nephropathy, focal segmental necrotizing glomerulonephritis, and focal segmental glomerulosclerosis) [8]; and tubulointerstitial nephritis as a complication of drug exposure [9]. In addition, long-term treatment with antirheumatic drugs or non-steroidal anti-inflammatory drugs (NSAIDs) may adversely affect kidney function [7]. Previous studies have reported that approximately 7% of patients with JIA develop hypertension during follow-up [7], a prevalence higher than the 4% estimated for the general pediatric population [10]. This has been associated with the duration of disease activity, prolonged NSAID use—which can inhibit renal prostaglandins, leading to renal hypoperfusion and subsequent hypertension—and methotrexate treatment, which may have potential nephrotoxic effects [7]. This could create a vicious cycle in which hypertension and kidney injury mutually exacerbate each other.

To the best of our knowledge, no studies have specifically investigated acute kidney injury (AKI) in children with JIA. Only a recent study reported an AKI prevalence of 1.8% in children with inflammatory arthritis [11]. However, this study also included ANCA-associated vasculitis and lupus, conditions characterized by primary kidney involvement [12, 13].

As per other pediatric illnesses [14–22], we hypothesized that a significant number of patients with JIA may experience AKI at the diagnosis of their chronic arthritis. We also hypothesized that AKI could influence the subsequent risk of developing hypertension or chronic kidney disease (CKD). Based on this premise, we aimed to assess the prevalence and risk factors of AKI at JIA diagnosis (primary outcome), as well as to evaluate its impact on the risk of CKD and hypertension during follow-up (secondary outcome).

## Methods

In this multicenter study, we retrospectively enrolled all children attending our pediatric rheumatology units between January 1998 and January 2025 with a diagnosis of JIA. Written informed consent was obtained from all data subjects prior to any clinical procedure, and the Research Ethics Committee of the University of Campania “Luigi Vanvitelli” approved this retrospective study.

Inclusion criteria were (i) diagnosis of JIA established according to the International League Against Rheumatism (ILAR) criteria [23]; (ii) a minimum of two follow-up visits within a 1-year interval; and (iii) age < 18 years at diagnosis of JIA. Exclusion criteria were (i) refusal to undergo the follow-up and investigations we proposed, in line with our clinical practice; (ii) missing data; (iii) loss to follow-up; (iv) known primary kidney disease; (v) other underlying diseases; (vi) used ACE inhibitors, angiotensin receptor blocker, and aminoglycosides before JIA diagnosis; and (vii) features of “COPA” syndrome.

Patients diagnosed with JIA before the introduction of the ILAR criteria were included only if their clinical charts provided evidence that they met the ILAR diagnostic criteria at the time of diagnosis [23].

None of the patients showed features of or were diagnosed with COPA syndrome (autosomal dominant disease characterized by lung disease, arthritis, and glomerulonephritis that can mimic JIA) during follow-up.

### Follow-up schedule

Patient follow-up was conducted in accordance with standard clinical practice at the participating centers. Individuals with a newly established diagnosis of JIA were evaluated monthly until clinical remission was achieved; subsequent visits were scheduled every 3 or 6 months according to disease activity and ongoing treatment.

At each visit, clinical data—including height, weight, body mass index, blood pressure, and presence and duration of morning stiffness—were recorded, together with laboratory parameters such as complete blood count, hemoglobin, platelet count, serum creatinine, blood urea nitrogen, glucose, electrolytes (sodium, potassium, chloride), liver enzymes (aspartate and alanine aminotransferase), C-reactive protein (CRP), procalcitonin, erythrocyte sedimentation rate (ESR), urine dipstick analysis, and the albumin-to-creatinine ratio (ACR). Antinuclear antibodies (ANA) were assessed at the time of JIA diagnosis and subsequently on a yearly basis.

Serum creatinine was measured using an IDMS-traceable assay, beginning in 2009 at one center and in 2019 at the other. Before these time points, the Jaffe method was used. Blood samples were obtained after overnight fasting, and first-morning urine samples were routinely collected for analysis.

Overall disease activity was quantified using the Juvenile Arthritis Disease Activity Score based on 10 joints (JADAS-10). This composite index incorporates the count of active joints (range 0–10), the physician’s global assessment of disease activity measured on a 10-cm visual analog scale (where 0 indicates no activity and 10 indicates maximum activity), and the parent’s or patient’s global assessment of well-being (10-cm VAS, where 0 indicates “very well” and 10 indicates “very poor”) [24]. The ESR was included after normalizing to a 0–10 scale using a standardized formula: (ESR[mm/hour] − 20)/10 [24]. The estimated glomerular filtration rate (eGFR) was calculated using the Hoste (age) equation [25] for creatinine measured with the IDMS-traceable method and the Schwartz equation for creatinine measured using the Jaffe method [25, 26]. A summary of all variables collected for the study is provided in Table 1.

**Table 1:** Clinical and laboratory characteristics of all enrolled patients, and of the patients with and without AKI

	All patients No. = 192	AKI (yes) No. = 45	AKI (no) No. = 147	*p*
Age at the onset, yr, mean (SDS)	6.7 (4.1)	4.8 (2.6)	7.2 (4.2)	<0.001
Male gender, No. (%)	56 (29.2)	13 (28.9)	43 (29.3)	0.96
Birth weight, gr, mean (SDS)	2363.8 (118.5)	2673.8 (1414.6)	2266.8 (1588.6)	0.14
Small for gestational age, No. (%)	17 (8.9)	15 (10.2)	2 (4.4)	0.37
Gestational age, weeks, median (IQR)	39 (1)	39 (2)	38 (2)	0.46
Preterm birth, No (%)	13 (6.8)	2 (4.4)	11 (7.5)	0.74
NSAIDs before JIA diagnosis, No. (%)	68 (35.4)	18 (40.0)	60 (34.0)	0.46
Weeks of NSAIDs treatment before JIA diagnosis, median (IQR)	1.0 (3.0)	1.0 (3.0)	1.0 (2.0)	0.50
Cumulative dose of NSAIDs before JIA diagnosis, mg/kg, median (IQR)	2.1 (7.0)	2.1 (8.0)	2.1 (7.0)	0.64
Number of active joints, mean (SDS)	3.1 (0.21)	3.0 (3.3)	3.1 (2.7)	0.84
Maximal body temperature, °C, mean (SDS)	36.8 (0.6)	37.0 (0.8)	36.8 (0.8)	0.09
Refill time > 2 s	1 (0.5)	0 (0)	1 (0.7)	0.99
Peak serum creatinine levels, mean (SDS)	0.47 (0.16)	0.60 (0.16)	0.42 (0.14)	<0.001
Highest-to-basal creatinine ratio, mean (SDS)	1.2 (0.39)	1.95 (0.38)	1.1 (0.20)	<0.001
eGFR, mL/min/1.72 m^2^, mean (SDS)	103.6 (29.7)	68.4 (16.2)	114.4 (24.1)	<0.001
Serum Urea levels, mg/dL, median (IQR)	27.9 (1.5)	21 (18)	27 (11)	0.14
Proteinuria at JIA diagnosis^a^, No. (%)	4 (2.1)	3 (6.8)	1 (0.7)	0.04
ESR, mm/h, mean (SDS)	38.5 (2.3)	44.4 (33.7)	36.5 (29.8)	0.14
CRP, mg/L, median (IQR)	36.0 (69.5)	134.7 (186.4)	21.8 (56.0)	0.01
Procalcitonin^b^, ng/mL, median (IQR)	0.08 (0.32)	1.2 (2.17)	0.06 (0.06)	<0.001
Ferritin, ng/mL, median (IQR)	126.7 (274.6)	468.0 (1748.6)	91.9 (101.5)	0.02
Hematocrit, %, median (IQR)	34.5 (6.2)	31 (6.5)	34.5 (4.9)	0.21
Hemoglobin, gr/dL, median (IQR)	11.4 (2.0)	10.6 (3.0)	11.4 (2.0)	0.03
Platelets, n/mcL, median (IQR)	397.0 (184)	554.0 (439)	395.5 (122)	0.36
WBC, n/mcL, median (IQR)	9970.0 (7880.0)	16,200 (13,225)	9590 (6065)	0.06
Neutrophils, n/mcL, median (IQR)	5120.0 (8555)	12,080 (9513)	4720 (3660)	0.02
Serum sodium levels, mEq/L, median (IQR)	138.0 (4.0)	136.0 (5.0)	138.0 (3.0)	0.89
Serum chloride levels, mEq/L, median (IQR)	100.0 (5.0)	99.0 (4.0)	102.0 (4.0)	0.86
Serum potassium levels, mEq/L, mean (SDS)	4.4 (0.03)	4.4 (0.41)	4.4 (0.42)	0.6
Serum phosphates levels, mg/dL, mean (SDS)	4.8 (0.06)	4.8 (0.8)	4.7 (0.6)	0.75
Serum calcium levels, mg/dL, mean (SDS)	9.7 (0.03)	9.7 (0.43)	9.7 (0.37)	0.80
RF+, No. (%)	9 (4.7)	1 (2.2)	8 (5.4)	0.68
ANA+, No. (%)	90 (46.9)	29 (64.4)	61 (41.5)	0.007
Persistent oligoarthritis, No. (%)	108 (56.3)	25 (55.6)	83 (56.4)	0.91
Extended oligoarthritis, No. (%)	13 (6.8)	1 (2.2)	12 (8.2)	0.31
RF− polyarthritis, No. (%)	29 (15.1)	8 (17.7)	21 (14.3)	0.57
RF+ polyarthritis, No. (%)	14 (7.2)	5 (11.1)	9 (6.1)	0.32
Enthesitis related arthritis, No. (%)	7 (3.6)	0 (0)	7 (4.8)	0.20
Psoriatic arthritis, No. (%)	13 (6.8)	3 (6.7)	10 (6.8)	0.97
Systemic arthritis, No. (%)	8 (4.2)	3 (6.7)	5 (3.4)	0.39
VAS, mean (SDS)	5.8 (0.15)	5.6 (1.9)	5.8 (2.0)	0.49
JADAS-10, mean (SDS)	16.3 (0.58)	16.8 (8.5)	16.1 (7.2)	0.61
Uveitis, No. (%)	21 (10.9)	6 (13.3)	15 (10.2)	0.58
CAKUT, No. (%)	8 (4.2)	3 (7.3)	5 (3.6)	0.39
Age at last follow-up, yr, mean (SDS)	12.4 (6.4)	11.9 (6.8)	12.6 (6.3)	0.54
Follow-up duration, yr, mean (SDS)	6.3 (5.5)	5.9 (5.1)	7.6 (6.3)	0.06
Utilization of methotrexate + NSAIDs^c^ after JIA diagnosis, No. (%)	86 (44.8)	18 (40.0)	68 (44.8)	0.46
Months of NSAIDs^c^ treatment after JIA diagnosis, median (IQR)	36 (48)	42 (110)	30 (48)	0.96
Cumulative dose of NSAIDs^c^ after JIA diagnosis, mg/kg, median (IQR)	19,800 (29,400)	20,700 (39,150)	18,000 (28,800)	0.74
Months of methotrexate treatment after JIA diagnosis, median (IQR)	30 (43)	31 (48)	30 (43)	0.86
Cumulative dose of methotrexate after JIA diagnosis, mg/m^2^, median (IQR)	1500 (2160)	1320 (2580)	1500 (2160)	0.47
KD at last follow-up, No. (%)	23 (12.0)	13 (28.9)	10 (6.8)	<0.001
Hypertension at last follow-up, No. (%)	6 (3.1)	4 (8.9)	2 (1.4)	0.028
Reduced eGFR at last follow-up, No. (%)	18 (9.4%)	10 (22.2)	8 (5.4)	<0.001
Proteinuria at last follow-up, No. (%)	1 (0.52)	1 (2.2)	0 (0)	0.23

^a^This data was available for 95 patients

^b^This data was available for 46 patients

^c^NSAIDs were administered only after resolution of AKI and only when clinically indicated

For normal distributed variables, means ± SDS are shown, while for non-parametric ones, median and lower and upper quartiles are shown

*AKI* acute kidney injury, *ANA* antinuclear antibodies, *CAKUT* congenital anomalies of the kidney and urinary tract, *CRP* C-reactive protein, *eGFR* estimated glomerular filtration rate, *ESR* erythrocyte sedimentation rate, *IQR* interquartile range, *JADAS-10* juvenile arthritis disease activity score based on 10 joints, *JIA* juvenile idiopathic arthritis, *KD* kidney damage, *RF* rheumatoid factor, *SDS* standard deviation score, *VAS* visual analog scale

### Treatment of JIA

In our cohort, JIA treatment was assigned according to recommendations of the American College of Rheumatology [27]. NSAIDs and intra-articular corticosteroids were prescribed as first-line treatments for oligoarticular JIA. Disease-modifying antirheumatic drugs (DMARDs), such as methotrexate, were prescribed for patients with polyarticular JIA or for those with oligoarticular disease unresponsive to NSAIDs or intra-articular corticosteroids [27].

Biological drugs were prescribed for patients with systemic JIA and for those with polyarticular or oligoarticular JIA unresponsive to methotrexate or in cases of methotrexate intolerance. In our centers, biologics have been used to treat JIA since 2001. Systemic corticosteroids were administered for short courses in patients with systemic JIA or with high disease activity to achieve remission [27]. The available treatments for JIA are summarized in Table 2.

**Table 2:** Main drugs used in JIA treatment

Drugs	Mechanism of action	Indications
NSAIDs (ibuprofen or naproxen)	Inhibition of cyclo-oxygenase, which reduces prostaglandin synthesis in various tissues including the synovial fluid	To reduce inflammation in non-severe oligoarticular JIA
Steroids (intra-articular or systemic)	They inhibit inflammation by reducing the production of pro-inflammatory mediators such as prostaglandins and leukotrienes, and by immunosuppressing the immune response by blocking the action of inflammatory cells and cytokines	Intra-articular steroids: immediate action in absence of systemic side effect, useful in oligoarticular JIA Systemic steroids: very effective in systemic JIA to reduce inflammation
Methotrexate	Folic acid antagonist that blocks RNA and DNA synthesis Specifically inhibits the proliferation of high-turnover cells	Oligo/polyarticular JIA not responding to NSAIDs or intra-articular steroids
Etanercept (anti–TNF-α)	Chimeric fusion protein that binds soluble TNF-α and TNF-β (lymphotoxin), rendering them biologically inactive	Oligo/polyarticular JIA not responding to methotrexate or methotrexate intolerance
Adalimumab (anti–TNF-α)	Recombinant human IgG1 monoclonal antibody specific for human TNF-α It binds persistently and irreversibly to TNF-α	Oligo/polyarticular JIA not responding to methotrexate or methotrexate intolerance
Tocilizumab (anti–IL-6)	Humanized monoclonal antibody Binds to the IL-6 receptor, blocking the cytokine’s binding	Oligo/polyarticular JIA not responding to methotrexate or anti–TNF-α drugs or systemic JIA
Abatacept (anti–CTLA-4)	Fusion protein that binds to the costimulatory molecules of antigen-presenting cells, blocking their interaction with CD28 on T cells	Oligo/polyarticular JIA not responding to methotrexate or anti–TNF-α drugs
Anakinra (anti–IL-1)	Recombinant form of the IL-1 receptor antagonist. It acts selectively by blocking the action of IL-1	Systemic JIA
Canakinumab (anti–IL-1)	A human monoclonal antibody that selectively binds to IL-1β, blocking its activity	Systemic JIA

*IL-1* interleukin 1, *IL-6* interleukin 6, *CTLA-4* cytotoxic T-lymphocyte-associated protein 4, *DNA* deoxyribonucleic acid, *JIA* juvenile idiopathic arthritis, *NSAIDs* non-steroidal anti-inflammatory drugs, *RNA* ribonucleic acid, *TNF-α* tumor necrosis factor alpha, *TNF-β* tumor necrosis factor beta

In patients who developed AKI after NSAID exposure, NSAID therapy was discontinued and corticosteroids were initiated, either as intra-articular injections or oral prednisone at a dose of 1 mg/kg for a short course, depending on the JIA subtype. Subsequently, DMARDs were started.

### Primary outcome definition

The primary endpoint of the study was AKI, which was identified in accordance with the Kidney Disease: Improving Global Outcomes (KDIGO) guidelines based on serum creatinine criteria [28]. Baseline creatinine values were derived using previously validated back-estimation approaches [29]. As previously described, height-dependent and height-independent basal serum creatinine estimation methods were comparable [29]. Therefore, we applied the Hoste (age) equation [25] to back-calculate baseline serum creatinine, assuming median age-based estimated glomerular filtration rate (eGFR) values for children ≤ 2 years [30] and eGFR = 120 mL/min/1.73 m^2^ for those >2 years [30]. AKI staging was defined as follows: (i) no AKI: all serum creatinine values < 1.5 × baseline; stage 1 AKI: 1.5 to <2 × baseline; stage 2 AKI: 2 to <3 × baseline; stage 3 AKI: ≥3 × baseline.

### Secondary outcome definition

The secondary outcome was the occurrence of CKD and/or hypertension (combined under the term “Kidney damage, KD” in this manuscript) at follow-up. CKD was defined as either: (i) ACR ≥ 30 mg/g persisting for >3 months, confirmed in three separate samples; or (ii) eGFR < 90 mL/min/1.73 m^2^ for >3 months [31].

To simplify the reading of the manuscript, although an ACR between 30 and 300 mg/g indicates microalbuminuria, in this manuscript we defined an ACR ≥ 30 mg/g as proteinuria.

Blood pressure was recorded after 15 min of seated rest. Hypertension in this study was defined retrospectively following the methods described by Flynn et al. [32, 33]. According to these guidelines, we used the mean of three auscultatory readings only when initial oscillometric measurements were elevated. Hypertension was diagnosed if elevated auscultatory readings were recorded on three different occasions. All patients identified as hypertensive received standard follow-up assessments for JIA, including evaluation for lipid profile, hemoglobin A1c, plasma renin activity, and kidney ultrasound [34].

### Statistical analysis

Statistical significance was defined as a *p* value below 0.05. Continuous variables were compared using an independent-samples *t*-test when normally distributed, or the Mann–Whitney test for non-normal distributions. Categorical variables were compared using the chi-squared test.

#### Primary outcome

Logistic regression models were applied to explore associations with AKI. Parameters showing a significant association with AKI (*p* < 0.05) in the initial comparison between patients with and without AKI (Table 1) were included in the univariate logistic regression analysis. Proteinuria at JIA diagnosis was excluded from the logistic regression models because it was observed in only four patients.

Variables with *p* < 0.05 in the univariate analysis were entered into the multiple logistic regression model. Procalcitonin was excluded from the multiple analysis due to availability in only 46 patients, while ferritin was excluded because of collinearity with CRP and a wider confidence interval compared to CRP. For the multiple logistic regression, the significance threshold was adjusted using Bonferroni correction and set at *p* < 0.017.

#### Secondary outcome

The secondary outcome was analyzed using Kaplan–Meier survival methods. Time-to-event was calculated from the date of hospital admission for JIA diagnosis. Differences between survival curves were assessed with the log-rank test. Cox regression analysis was used to calculate the hazard ratio (HR) for the secondary outcome according to AKI status at JIA diagnosis, adjusting for sex, cumulative dose and duration of methotrexate and NSAIDs treatment, low birth weight, and preterm birth. We also calculated the odds ratio (OR) for developing the secondary outcome in patients with AKI at JIA diagnosis, adjusted for sex, age at last follow-up, disease duration, cumulative dose and duration of methotrexate and NSAIDs treatment, low birth weight, and preterm birth. NSAID and methotrexate exposure were not analyzed as primary determinants of KD, since their association with KD in children with JIA has already been established in a previous study [7]. Accordingly, in the present analysis, NSAIDs and methotrexate were included as covariates to adjust the association between AKI and long-term KD in both Cox and logistic regression models in children with JIA. We did not adjust these models for the use or cumulative dose of biological agents because these factors have not previously been associated with the development of KD [7].

All statistical analyses were performed using Stat-Graph XVII software for Windows, except for the Kaplan–Meier analysis, which was conducted with GraphPad Prism 7 software for Windows.

## Results

### General characteristics

A total of 209 patients were eligible for inclusion; 17 were excluded due to missing serum creatinine values at admission for JIA diagnosis. Consequently, 192 patients (29.2% male) with a mean age of 6.7 ± 4.1 years (range: 0.6–17 years) met the inclusion criteria. Of these, 108 had persistent oligoarthritis, 13 extended oligoarthritis, 29 rheumatoid factor-negative polyarthritis, 14 rheumatoid factor-positive polyarthritis, 7 enthesitis-related arthritis, 13 psoriatic arthritis, and 8 systemic arthritis. The included and excluded patients showed similar characteristics (Supplementary Table 1).

### AKI at JIA diagnosis

Among the 192 patients, AKI was observed in 45 cases (23.4%). AKI prevalence was similar when comparing patients with JIA onset before (12/62 patients, 19.4%) and after (33/130 patients, 24.4%) the implementation of the IDMS-traceable method for measuring serum creatinine (*p* = 0.36). None required hemodialysis. Of the patients with AKI, one reached stage 3, nine reached stage 2, and 35 reached stage 1. In 28 cases, the maximum AKI stage was present at admission; in the remaining patients, it occurred within 1 day (*n* = 4), 2 days (*n* = 4), 3 days (*n* = 3), or between days 4 and 7 of hospitalization (*n* = 6). At the first follow-up, kidney function had normalized in all patients.

### KD at the last follow-up

After a mean follow-up of 6.3 years (5.5 SDS) with a range between 1 and 27.5 years, 23 patients (12%) presented with KD. In more detail, 19 patients presented with CKD (eGFR persistently <90 mL/min/1.73 m^2^ in 18 patients and persistent proteinuria in 1 patient), 4 hypertension, and 2 CKD and hypertension.

Among the six patients with hypertension, two had stage 2 hypertension (one in the AKI group and one in the non-AKI group), and four had stage 1 hypertension (three in the AKI group—one of whom was born small for gestational age—and one in the non-AKI group). All patients with stage 1 hypertension achieved good blood pressure control with ramipril. The patient with stage 2 hypertension in the non-AKI group, with obesity, was also well controlled with ramipril. The patient with stage 2 hypertension in the AKI group required a combination of ramipril and amlodipine to achieve blood pressure control. No additional causes of secondary hypertension were identified, apart from underlying JIA, concomitant medications, and the above-mentioned risk factors. The evaluation performed in hypertensive patients as part of the screening for other secondary causes was normal in all cases.

The cumulative proportion of patients free from KD before and after the introduction of biologic therapies for JIA was similar (62.5% at 23 years from JIA diagnosis among patients enrolled after and 71.1% at 27 years among those enrolled before the implementation of biologics in clinical practice; *p* = 0.06).

### Comparison of the patients with and without AKI

Compared with patients without AKI, those with AKI were younger and had a higher prevalence of proteinuria and ANA positivity, higher levels of CRP, procalcitonin, ferritin, and neutrophils, and lower hemoglobin levels (Table 1). Moreover, at the last follow-up, patients who had experienced AKI showed a higher prevalence of KD overall—and specifically, higher rates of hypertension and reduced eGFR (Table 1).

### Logistic regression analysis

#### Factors associated with AKI development

In the univariate analysis, age, CRP, procalcitonin, ferritin, and ANA positivity were significantly associated with AKI development (Table 3). In the multiple logistic regression analysis, age, CRP, and ANA positivity remained significantly associated with AKI development (Table 3).

**Table 3:** Exploratory logistic regression analysis of risk factor potentially associated with AKI

Risk factors	Univariate			Multiple		
	OR	95% CI	*p*	OR	95% CI	p*
Age^a^, yr	1.2	1.08–1.3	<0.001	1.2	1.1–1.3	0.002
CRP^b^, mg/L	1.1	1.008–1.14	0.028	1.1	1.03–1.2	0.007
Procalcitonin^c^, ng/mL	37.8	1.6–908.8	0.025	–	–	–
Ferritin^d^, ng/mL	1.06	1.006–1.12	0.031	–	–	–
Hemoglobin^e^, g/dL	1.3	0.99–1.6	0.06	–	–	–
Neutrophils^f^, n/mcL	1.1	0.99–1.2	0.07	–	–	–
ANA+	2.6	1.3–5.1	0.008	4.1	1.8–9.2	<0.001

*ANA* antinuclear antibodies, *AKI* acute kidney injury, *CRP* C-reactive protein

*Bonferroni-corrected significance threshold: *p* = 0.017

^a^1 year decrease in age

^b^10 mg/L increase in CRP levels

^c^1 ng/mL increase in PCT levels

^d^100 ng/mL decrease in serum ferritin levels

^e^1 g/dL decrease in serum Hb levels

^f^1000/mcL increase in neutrophils levels

#### Factors associated with KD at last follow-up

After adjusting for sex, age at last follow-up, disease duration, cumulative dose and duration of methotrexate and NSAIDs treatment, low birth weight, and preterm birth, AKI was associated with an OR of 5.8 (95% CI: 2.4–15.2; *p* < 0.001) for developing KD at the last follow-up.

### Kaplan–Meier analysis of the development of KD at the last follow-up

**Fig. 1:**
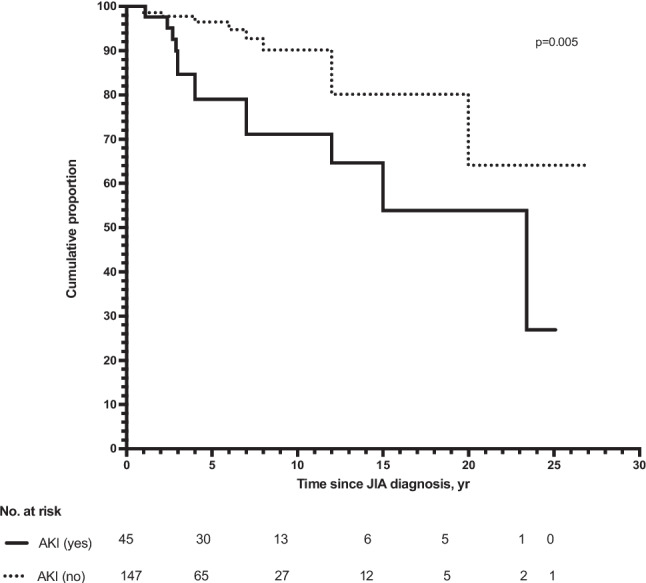
Kaplan–Meier analysis of the development of KD at the last follow-up. The cumulative proportions of KD onset for patients having developed AKI at JIA diagnosis at the end of the following intervals of follow-up were as follows: 1 year, 100%; 5 years, 79%; 10 years, 71.1%; 15 years, 53.9%; 20 years, 53.9%; 25 years, 26.9%. The cumulative proportions of KD onset for patients not having developed AKI at JIA diagnosis at the end of the following intervals of follow-up were as follows: 1 year, 98.6%; 5 years, 96.5%; 10 years, 90.2%; 15 years, 90.2%; 20 years, 64.1%; 25 years, 64.1%

We separately analyzed the Kaplan–Meier curves for subjects with and without AKI at JIA diagnosis. The cumulative proportion remaining free from KD at 25.1 years of age was 26.9% among those who had developed AKI and 64.1% among those without AKI at JIA diagnosis (*p* = 0.005) (Fig. 1). In an exploratory analysis of patients with AKI, KD-free survival did not differ according to AKI stage (log-rank *p* = 0.62). The cumulative proportion of patients free from KD was 0% at 24 years in stage 2–3 AKI and 37.4% at 27 years in stage 1 AKI.

In the exploratory Cox regression analysis (adjusted for sex, low birth weight, cumulative dose and duration of methotrexate and NSAIDs treatment, and preterm birth) of factors potentially associated with KD, a history of AKI at JIA diagnosis was associated with a HR of 3.7 (95% CI: 1.9–8.9; *p* = 0.002).

## Discussion

This study analyzed kidney outcomes in a multicenter cohort of children with JIA. We found that approximately one quarter of children at JIA diagnosis experienced AKI, although none required hemodialysis.

Only one previous study examined this aspect in a mixed population with inflammatory arthritis including rheumatoid arthritis, JIA, ankylosing spondylitis, inflammatory spondyloarthropathy, and other inflammatory arthritis [11]. AKI was reported in 89 of 4918 subjects (1.8%) using a national database [11]. This prevalence is markedly lower than the 23.4% AKI prevalence observed in our cohort. The difference may be due to the heterogeneity of conditions included in the study by Lin et al. [11], as well as to the fact that AKI was defined not by laboratory data but through International Classification of Diseases, Ninth and Tenth edition diagnostic codes in medical records [11]. Indeed, AKI, especially in its milder forms, often goes undetected in daily clinical practice [35].

In our study, factors independently associated with AKI after multivariable logistic regression were age, CRP levels, and ANA positivity. These findings provide interesting pathophysiological insights. In JIA, both the degree of inflammation (as indicated by CRP levels) and younger age were significantly associated with AKI development at disease diagnosis. Younger age may reflect a reduced physiological reserve during acute illness, including a lower capacity to compensate for fluid losses and hemodynamic changes, potentially increasing susceptibility to AKI [36]. Moreover, the association with ANA positivity is noteworthy. This may be related to the fact that ANA positivity could indicate more severe systemic involvement in JIA [37] and is also associated with higher mortality in the general population, particularly when concomitant with rheumatological diseases [38].

Therefore, as with community-acquired pneumonia, viral bronchiolitis, acute appendicitis, and febrile urinary tract infections, inflammation appears to play a pivotal role in the development of AKI in JIA [14, 16, 17, 19]. This could suggest that effective control of inflammation at the time of JIA diagnosis may be important to promote AKI resolution or to prevent its occurrence in patients who have not yet developed it. Similarly, prompt and appropriate management of potential future inflammatory relapses may help prevent AKI recurrence concomitant with disease flares. This is particularly relevant because AKI itself increases the risk of CKD [39], which is already elevated in patients with JIA [7].

Normal kidney function results from a balance between nephron number and kidney stress [20] and other pathophysiological mechanisms—such as low nephron mass, dehydration, or drug exposure—should also be considered in AKI pathophysiology [15, 19–21]. In fact, the coexistence of multiple mechanisms could further increase the risk of AKI [22].

In a previous single-center cohort study, it was shown that about 8% of children with JIA develop KD (hypertension and/or CKD) [7]. The main risk factor was longer exposure to both NSAIDs and methotrexate, reflecting a more severe form of the disease [7]. Other data indicate that subjects with JIA demonstrate reduced eGFR, increased urinary albumin excretion, and a higher renal resistive index, which is associated with renal parenchymal damage, compared with controls [40].

Evidence also indicates that experiencing AKI increases the risk of developing KD later in life, with the risk rising significantly with increasing AKI stage [39]. However, in an exploratory analysis among patients with AKI, we did not observe a statistically significant association between AKI severity and long-term KD-free survival, although patients with stage 2–3 AKI appeared to develop KD earlier than those with stage 1 AKI. This finding is likely related to the very limited number of patients in each subgroup.

Leveraging the multicenter enrollment and long-term follow-up, we evaluated the impact of AKI at JIA diagnosis on the development of KD, adjusting for factors previously associated with this condition in the context of JIA [7], as well as for other determinants that can affect nephron number and thereby increase the risk of KD, such as gestational age and birth weight [41].

Interestingly, we found that about 12% of patients with JIA developed KD during follow-up, confirming previous findings [7]. It should be noted that one patient with hypertension was obese, a condition that may have contributed to elevated blood pressure. However, hypertension in children is often multifactorial, and the presence of additional risk factors does not preclude a contributory role of JIA-related inflammation or treatment exposure.

Additionally, we provided new evidence on the role of AKI at JIA diagnosis in increasing the risk of future KD. The cumulative proportion remaining free from KD at 25.1 years of age was 26.9% among those who had developed AKI, compared with 64.1% among those without AKI at JIA diagnosis (*p* = 0.005). These patients had a six-fold increased risk of KD in logistic regression and a four-fold increased risk in Cox regression.

In our population, eight patients had kidney ultrasound findings suggestive of congenital anomalies of the kidney and urinary tract (CAKUT). However, these anomalies were mild, incidental, and asymptomatic, and their prevalence did not differ between patients who did or did not develop AKI or KD.

This study has several limitations. First, the retrospective design represents an inherent limitation, precluding the establishment of definitive causal relationships between specific clinical factors and the development of AKI. Accordingly, our analyses focused on identifying factors independently associated with AKI rather than a single etiological cause. Second, the KDIGO urine output criteria for AKI were not applied because urine output data were unavailable, which may have led to an underestimation of AKI prevalence. Third, baseline serum creatinine was back-calculated using estimation methods because direct baseline measurements were not available. However, back-calculation of baseline serum creatinine is widely accepted and commonly used in pediatric AKI research. Moreover, serum creatinine has intrinsic limitations because it is influenced by age and muscle mass in children [42], and we did not have any measurements of cystatin C. Fourth, data on urinary microscopy were largely unavailable for most patients and therefore could not be included in the analyses. Fifth, potential temporal heterogeneity related to changes in JIA management and laboratory methods over the study period should be acknowledged. Biologic therapies were introduced in our centers in 2001, and a small number (*n* = 9) of patients were enrolled before their routine use. In addition, serum creatinine assays changed with the implementation of IDMS-traceable methods at different times across centers. Although no significant differences in kidney outcomes were observed according to these changes, a residual effect cannot be entirely excluded. Sixth, the wide range of follow-up duration, with shorter follow-up in some patients, may have affected the assessment of long-term kidney outcomes; therefore, time-to-event analyses were used to account for variable follow-up and censoring. Finally, no patient underwent 24-h ambulatory blood pressure monitoring; therefore, data on the prevalence of masked hypertension were not available.

In conclusion, approximately one quarter of children with JIA have AKI at presentation, mostly mild. Independent factors associated with AKI development were age, CRP levels, and ANA positivity. Even mild AKI significantly increased the risk of KD during follow-up. Our findings highlight the importance of careful monitoring of kidney function in these patients, both at JIA diagnosis and throughout follow-up. This highlights for all pediatric rheumatologists the importance of assessing kidney function at the time of JIA diagnosis and of monitoring blood pressure, kidney function, and proteinuria during follow-up visits. In children with AKI, this approach may help the managing clinician exercise greater caution when initiating medications and guide appropriate fluid management.

## Supplementary Information

Below is the link to the electronic supplementary material.

Graphical abstract (PPTX 143 KB)

Supplementary file2 (DOC 49.5 KB)

## Data Availability

The data sets used and/or analyzed during the current study are available from the corresponding author on reasonable request.
