# Supplementary material for: Prevalence and risk factors for acute kidney injury at the diagnosis of juvenile idiopathic arthritis in children and its long-term implications for kidney health
Source: Pediatr Nephrol. 2026 Mar 3;41(7):2259–68. doi: 10.1007/s00467-026-07222-9 (PMC13197239; doi:10.1007/s00467-026-07222-9)
Supplement: Supplementary file 1 — Graphical abstract (PPTX 143 KB) [file 467_2026_7222_MOESM1_ESM.pptx]

## Slide 1
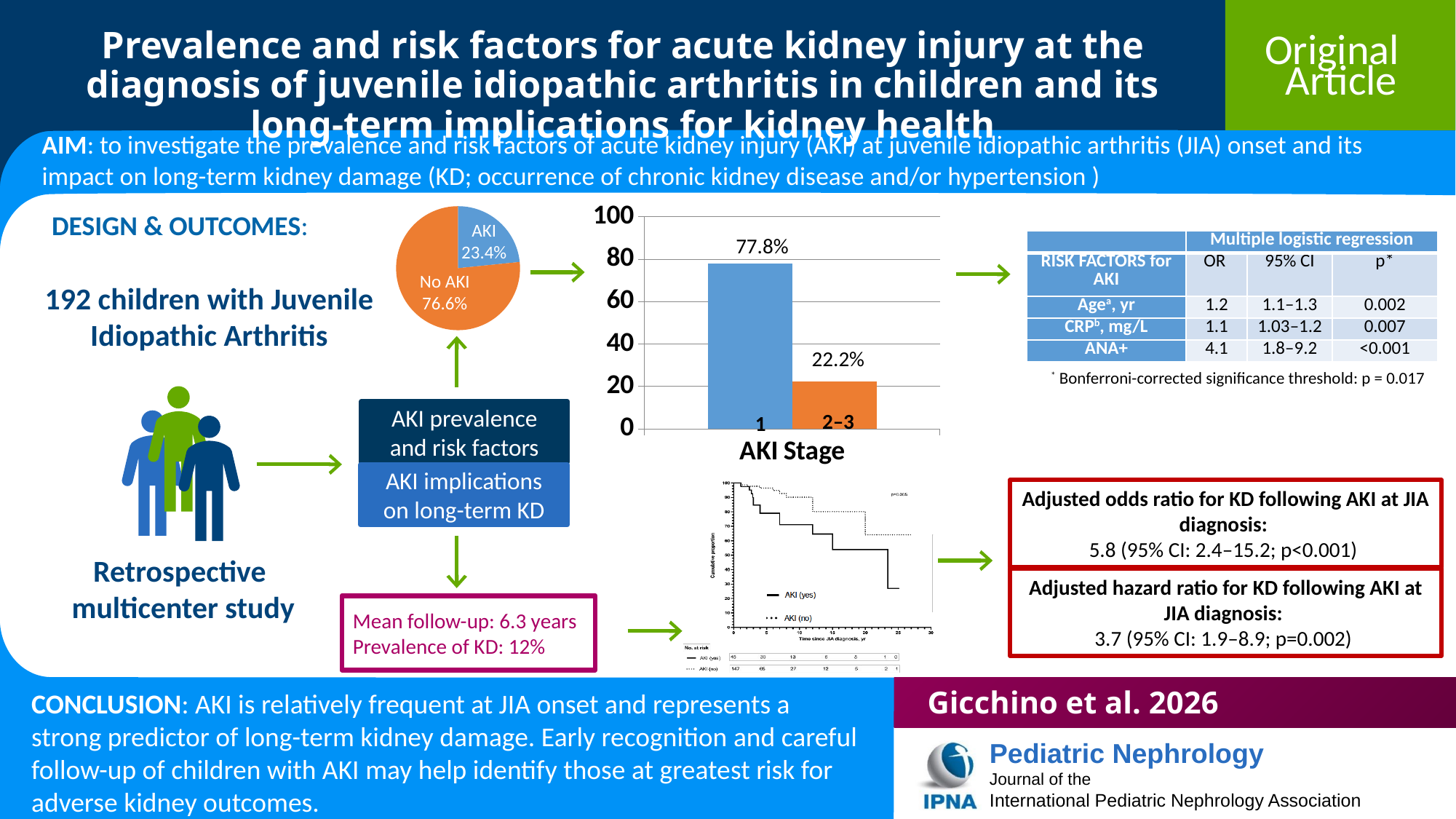

Prevalence and risk factors for acute kidney injury at the diagnosis of juvenile idiopathic arthritis in children and its long-term implications for kidney health
AIM: to investigate the prevalence and risk factors of acute kidney injury (AKI) at juvenile idiopathic arthritis (JIA) onset and its impact on long-term kidney damage (KD; occurrence of chronic kidney disease and/or hypertension )
### Chart
| Category | AKI prevalence |
|---|---|
| AKI | 23.4 |
| No AKI | 76.6 |AKI
23.4%
No AKI
76.6%
### Chart
| Category | 2 | 2–3 |
|---|---|---|
| AKI Stage | 77.8 | 22.2 |DESIGN & OUTCOMES:
77.8%
| | Multiple logistic regression | | |
| --- | --- | --- | --- |
| RISK FACTORS for AKI | OR | 95% CI | p\* |
| Agea, yr | 1.2 | 1.1–1.3 | 0.002 |
| CRPb, mg/L | 1.1 | 1.03–1.2 | 0.007 |
| ANA+ | 4.1 | 1.8–9.2 | <0.001 |
192 children with Juvenile Idiopathic Arthritis
* Bonferroni-corrected significance threshold: p = 0.017
22.2%
AKI prevalence and risk factors
AKI implications on long-term KD
2–3
1
Adjusted odds ratio for KD following AKI at JIA diagnosis:
5.8 (95% CI: 2.4–15.2; p<0.001)
Retrospective
multicenter study
Adjusted hazard ratio for KD following AKI at JIA diagnosis:
3.7 (95% CI: 1.9–8.9; p=0.002)
Mean follow-up: 6.3 years
Prevalence of KD: 12%
Gicchino et al. 2026
CONCLUSION: AKI is relatively frequent at JIA onset and represents a strong predictor of long-term kidney damage. Early recognition and careful follow-up of children with AKI may help identify those at greatest risk for adverse kidney outcomes.
