# Supplementary material for: Prevalence and risk factors for acute kidney injury at the diagnosis of juvenile idiopathic arthritis in children and its long-term implications for kidney health
Source: Pediatr Nephrol. 2026 Mar 3;41(7):2259–68. doi: 10.1007/s00467-026-07222-9 (PMC13197239; doi:10.1007/s00467-026-07222-9)
Supplement: Supplementary file 2 — Supplementary file2 (DOC 49.5 KB) [file 467_2026_7222_MOESM2_ESM.doc]

|  | **Included patients**  **No.= 192** | **Excluded patients**  **No.= 45** | **p** |
| --- | --- | --- | --- |
| **Age at the onset, yr, mean (SDS)** | 6.7 (4.1) | 7.6 (5.2) | 0.21 |
| **Male gender, No. (%)** | 56 (29.2) | 9 (20.0) | 0.21 |
| **Small for gestational age, No. (%)** | 17 (8.9) | 5 (11.1) | 0.63 |
| **Preterm birth, No (%)** | 13 (6.8) | 4 (8.9) | 0.62 |
| **NSAIDs before diagnosis, No. (%)** | 68 (35.4) | 15 (33.3) | 0.79 |
| **RF+, No. (%)** | 9 (4.7) | 3 (6.7) | 0.58 |
| **ANA+, No. (%)** | 90 (46.9) | 19 (42.2) | 0.69 |
| **Persistent oligoarthritis, No. (%)** | 108 (56.3) | 23 (51.1) | 0.53 |
| **Extended oligoarthritis, No. (%)** | 13 (6.8) | 2 (4.4) | 0.56 |
| **RF- polyarthritis, No. (%)** | 29 (15.1) | 8 (17.8) | 0.65 |
| **RF+ polyarthritis, No. (%)** | 14 (7.2) | 4 (8.9) | 0.71 |
| **Enthesitis related arthritis, No. (%)** | 7 (3.6) | 2 (4.4) | 0.80 |
| **Psoriatic arthritis, No. (%)** | 13 (6.8) | 3 (6.7) | 0.98 |
| **Systemic arthritis, No. (%)** | 8 (4.2) | 3 (6.7) | 0.47 |
| **Uveitis, No. (%)** | 21(10.9) | 4 (8.9) | 0.79 |
| **CAKUT, No. (%)** | 8 (4.2) | 1 (2.2) | 0.99 |
| **Age at last follow-up, yr, mean (SDS)** | 12.4 (6.4) | 13.5 (5.8) | 0.29 |
| **Follow-up duration, yr, mean (SDS)** | 6.3 (5.5) | 6.8 (5.1) | 0.58 |
| **Utilization of methotrexate+NSAIDs, No. (%)** | 86 (44.8) | 18 (40.0) | 0.56 |
| **KD at last follow-up, No. (%)** | 23 (12.0) | 4 (8.9) | 0.79 |
| **Hypertension at last follow-up, No. (%)** | 6 (3.1) | 1 (2.2) | 0.99 |
| **Reduced eGFR at last follow-up, No. (%)** | 18 (9.4%) | 3 (6.7) | 0.77 |
| **Proteinuria at last follow-up, No. (%)** | 1 (0.52) | 0 (0) | 0.99 |

**Supplementary Table 1. Clinical and laboratory characteristics of included and excluded patients**

For normal distributed variables means ± SDS are shown, while for non-parametric ones median and lower and upper quartiles are shown.

*Abbreviations:* AKI, acute kidney injury; ANA, antinuclear antibodies; CAKUT, congenital anomalies of the kidney and urinary tract; CRP, C-reactive protein; eGFR, estimated glomerular filtration rate; ESR, erythrocyte sedimentation rate; IQR, interquartile range; JADAS-10, juvenile arthritis disease activity score based on 10 joints; JIA, juvenile idiopathic arthritis; KD, kidney damage; RF, rheumatoid factor; SDS, standard deviation score; VAS, Visual Analogue Scale.
